# Fungal Empyema Thoracis Associated with *Clavispora lusitaniae*—First Report in a Domestic Cat

**DOI:** 10.3390/jof11030170

**Published:** 2025-02-20

**Authors:** Radka Garnoeva, Velina Dinkova

**Affiliations:** 1 Department of Veterinary Surgery, Faculty of Veterinary Medicine, Trakia University, 6000 Stara Zagora, Bulgaria; 2 Department of Veterinary Microbiology, Infectious and Parasitic Diseases, Faculty of Veterinary Medicine, Trakia University, 6000 Stara Zagora, Bulgaria; velina.dinkova@yahoo.com

**Keywords:** cat, pleural effusion, fungal empyema thoracis, *Clavispora lusitaniae*

## Abstract

Invasive fungal infections are life-threatening conditions that always pose a challenge to veterinary practitioners. The diagnostic and therapeutic approaches in a case of pleural effusion in a domestic cat with a 7-day history of progressive tachypnea were described. Fungal pyothorax was diagnosed on the basis of the clinical signs, radiography findings, complete blood counts, and isolation of the pathogen from pleural effusion samples on two occasions. After three thoracenteses for evacuation of the pleural exudate and 15-day therapy with terbinafine, the general condition of the patient was very good; the complete blood count and respiratory and heart rates returned to normal; and no diagnostic imaging signs of pleural effusion were present. To the best of our knowledge, this is the first report of empyema thoracis caused by *Clavispora lusitaniae* in a domestic cat. The described case emphasises the primary importance of timely identification of pathogenic agent(s) of feline pyothorax and appropriately prescribed treatment for the prevention of severe complications and fatal outcomes.

## 1. Introduction

Empyema thoracis is characterised by the accumulation of septic fluid within the pleural space. In cats, the aetiology of the condition often remains non-elucidated. Possible origins of infection include spread from an adjacent structure (bronchopneumonia, parapneumonic spread, oesophageal rupture), direct inoculation (migrating foreign body, penetrating injury, thoracic surgery), or haematogenous/lymphatic dissemination (sepsis) [1].

In most cases, the aetiology of feline pyothorax is associated with co-infection with obligate anaerobes (*Clostridium* spp., *Fusobacterium* spp., *Bacteroides* spp.) and/or facultative aerobes. The prevalence of cases with the participation of *Escherichia coli*, *Salmonella* spp., *Klebsiella* spp., *Pseudomonas* spp., etc., is less than 20% [2]. Fungal agents of pyothorax in cats are rarely reported and include *Cryptococcus* spp., *Candida albicans*, *Blastomyces dermatitidis* [3,4], and *Coccidioides* spp. [5].

As stated in a recent review on invasive fungal infections (IFIs) in cats, invasive candidiasis in this species is exceptionally rare, with less than 10 published cases, unlike non-invasive candidiasis of the lower urinary tract, which is more frequent [6]. IFIs may occur in cats of both sexes and at any age. Despite the fact that the disease history usually includes a co-morbidity, they are often diagnosed in apparently immunocompetent cats.

During the last years, the isolation of non-albicans *Candida* pathogens from invasive fungal infections and empyema, in particular, has increased [7,8]. Among them, *Clavispora lusitaniae*, formerly known as *Candida lusitaniae*, is an emerging nosocomial pathogen infecting immunosuppressed patients, and people on continuous antibiotic therapy and cancer chemotherapy [9], but it has also been reported as an agent of empyema thoracis in an immunocompetent patient with acute respiratory distress [10].

For the first time, invasive infection with *C. lusitaniae* in a cat was reported in a case of peritonitis [11]. The isolate was sensitive to tested antifungal drugs, including amphotericin B, which was attributed to the lack of previous selective pressure and isolation of the agent before the antifungal therapy.

The aim of the present report was to describe the diagnostic and therapeutic approach to the first known case of pyothorax associated with *Clavispora lusitaniae* in a domestic cat.

## 2. Case Presentation

A European shorthair cat was referred as an emergency case to the University Veterinary Hospital (UVH), Faculty of Veterinary Medicine, Trakia University, for examination and treatment. The cat was a 3-year-old intact male with a body weight of 3.2 kg. It was kept outdoor in the owners’ house yard, was a very skilled hunter of birds (sparrows), and in the past had been treated several times for bite and scratch wounds during the breeding season. The owners had noticed changes in the cat’s usual behaviour (decreased activity and poor appetite) for about 2–3 weeks. Seven days ago, the cat was brought to a private clinic with mild dyspnoea and cough and was diagnosed with catarrhal pneumonia. A treatment with 12.5 mg/kg amoxicillin–clavulanic acid (Synulox 50 mg palatable tablets, Zoetis, Leatherhead, UK) was prescribed, but despite that, the dyspnoea persisted and had worsened several hours before the referral to the UVH.

The initial physical examination revealed inspiratory dyspnoea, light pink mucous membranes, a capillary refill time (CRT) of 3 s, a rectal temperature of 38.6 °C, lethargy, and anorexia. Lung auscultation detected muffled heart sounds bilaterally (almost undetectable from the left), accelerated bronchovesicular breath sounds, visible rightward cardiac pulsations, tachypnea (67 min^−1^), and tachycardia (188 min^−1^). During the examination, no skin wounds were found, but the animal had cicatrices from previous injuries.

**Figure 1:**
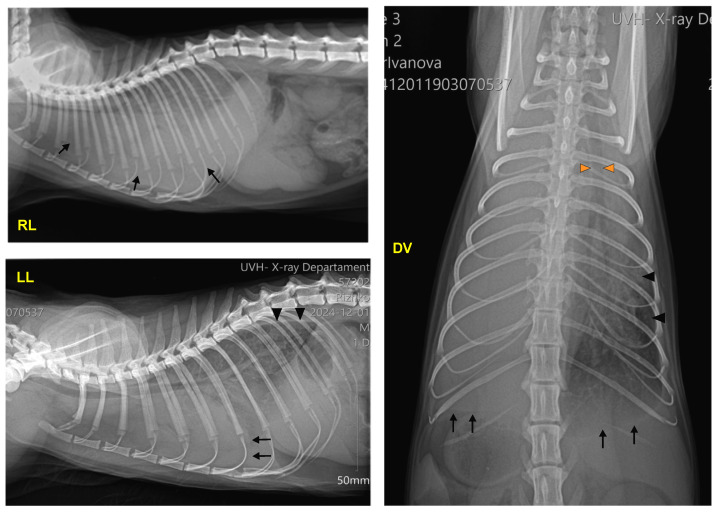
Right lateral (RL), left lateral (LL), and dorsoventral (DV) radiographs of the patient before the thoracentesis, demonstrating accumulated fluid (arrows; RL view), retraction of the lung lobes (black arrowheads, LL and DV views), pleural fissure lines (arrows, LL and DV views), and displacement of the trachea (orange arrowheads, DV view).

After the physical examination, three chest radiographs were taken in the left lateral, right lateral, and dorsoventral views with stationary radiography equipment (Philips, Bucky Diagnost CS4, Amsterdam, The Netherlands), the iQ-CR ACE digitiser system and iQ-VIEW/PRO version 2.7 software. The exposure data were 50 kVp and 10 mAs. The radiographs showed a bilateral pleural effusion with a clear horizontal boundary. The heart, the mediastinum, and the cranioventral and middle lung lobes on the right lateral view were obscured by the collected fluid. The left lateral view visualised a retraction of the lung lobes away from the dorsal chest wall, radiopaque interlobar fissure lines, rounding of lung margins, and moderate atelectasis. On the dorsoventral view, the trachea was displaced to the right side, and the left lung lobes were obscured by the exudate (Figure 1).

**Figure 2:**
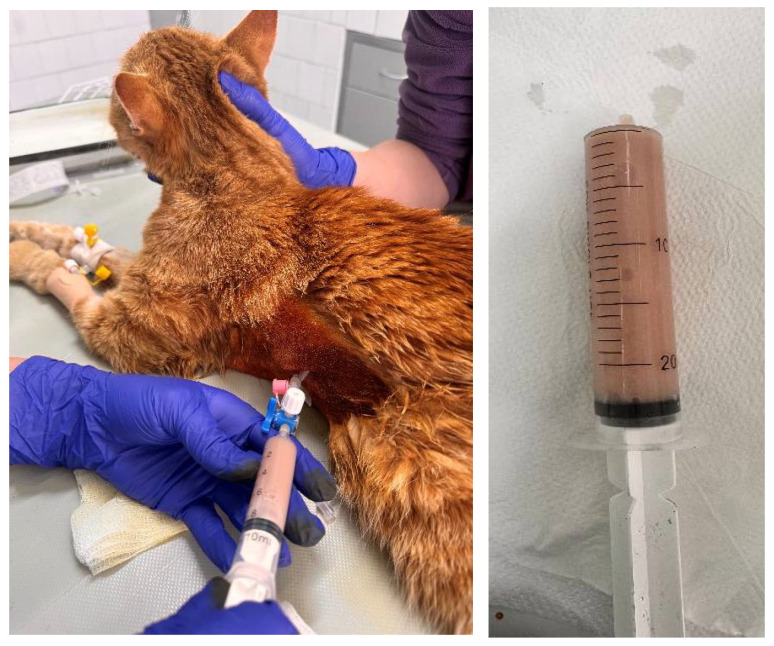
Evacuation of pleural fluid from the left-side 8th intercostal space (**left**) and gross appearance of the exudate (**right**).

Immediately after the radiography, the cat was anaesthetised with propofol (Propofol Fresenius^®^, Fresenius Kabi GmbH, Bad Homburg, Germany) at a dose of 15 mg/kg, applied intravenously. Fluid therapy included Ringer lactate (Ringer Braun, B. Braun Melsungen AG, Germany) at a flow rate of 10 mL/kg/hour. Bilateral thoracentesis was performed with a peripheral venous catheter and three-way stop cock (Figure 2). On the right side, the thoracentesis was made in the 6th–7th intercostal space, but only a small amount of fluid was removed. On the left side in the 8th intercostal space, 210 mL of opaque milky blood-tinged pleural fluid was evacuated. The fluid was odourless and viscous and contained flocculent matter.

Following the thoracentesis, the lateral radiography was repeated to evaluate the results after pleural fluid evacuation (Figure S1). Some fluid was still present in the cranioventral part of the chest, but the visualisation of organs was significantly improved. The cardiac silhouette was already visible, and the lung borders were more distinct.

The rapid blood tests for feline leukaemia virus (FeLV) and feline immunodeficiency virus (FIV) were negative. A blood sample was collected from the cephalic vein for complete blood count (CBC) and biochemical analysis. The results showed leukocytosis with total leukocyte counts of 46.47 G/L [reference range 5.5–19.5 G/L] and neutrophilia of 41 G/L [reference range 2.5–12.9 G/L]. The deviations in biochemical parameters included increased creatine kinase activity—2554 U/L [reference range 70–160 U/L]; increased serum total protein—84.4 g/L [reference range 57–80 g/L]; and increased globulins—52 g/L [reference range 28–45 g/L].

**Figure 3:**
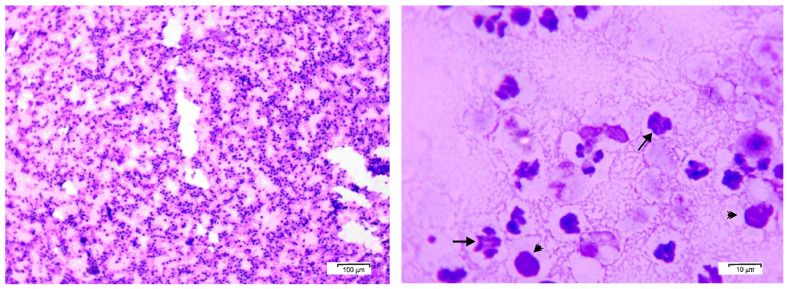
Cytological smear from the pleural fluid demonstrating lymphocytes, degenerated neutrophils (arrows), and macrophages (arrowheads). May–Grünwald Giemsa staining; scale bar 100 μm (**left**) and 10 μm (**right**).

Smears of the pleural fluid were prepared for cytological examination and stained with May–Grünwald Giemsa. The cytology showed predomination of lymphocytes of 5–10 μm in diameter. A large part of these cells had irregular nuclei, possibly due to their long stay in the exudate into the thorax. Neutrophils and macrophages were also visible (Figure 3).

A different antibiotic—clindamycin (Clindavet 75 mg, Provet, Attiki, Greece) at a dose of 5.5 mg/kg every 12 h for 7 days—was prescribed to replace the amoxicillin/clavulanic acid.

A sample for microbiological examination was drawn in a sterile 2 mL syringe and analysed immediately after submission at the microbiology lab. The exudate sample was inoculated on solid nutrient media: blood agar, McConkey agar, Sabouraud agar with 4% dextrose and 0.05 g/L chloramphenicol, and liquid nutrient media—tryptic soy broth (TSB) and thioglycollate medium at 37 °C.

**Figure 4:**
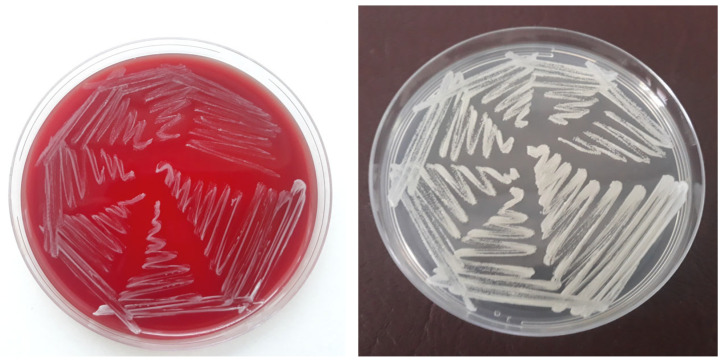
Growth of the pathogen 48 h after subculturing on blood agar at 37 °C (**left**) and Sabouraud dextrose agar at 37 °C (**right**).

**Figure 5:**
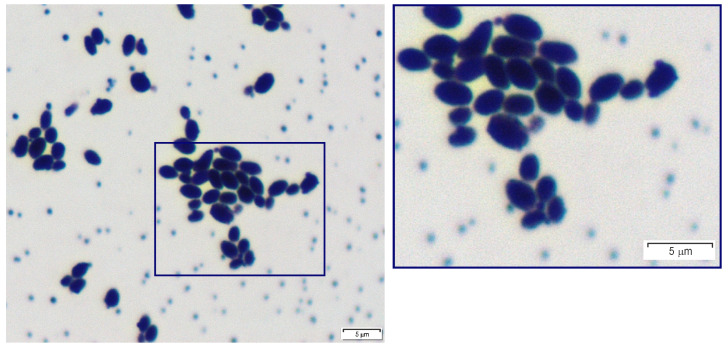
(**Left**): Gram-stained smear from a 24 h single colony subcultured on blood agar; (**Right**): enlarged view of rectangular area shows budding Gram-positive yeast cells. Scale bar = 5 μm.

By the 24th hour of cultivation, there was no visible growth on the solid nutrient media. A TSB sample was subcultured on blood agar. By the 48th hour, barely perceptible small white non-haemolytic colonies appeared on the blood agar plate, together with tiny white colonies on the Sabouraud agar. No growth was detected on the McConkey agar and on the 24 h blood agar subculture. By the 72nd hour, blood agar and Sabouraud dextrose agar plates demonstrated visible growth of numerous round, smooth, white non-confluent colonies (Figure 4). At that time, no growth was detected on McConkey agar, blood agar subculture and the thioglycollate medium. Gram-stained microscopic preparations of separate colonies visualised large rounded Gram-positive yeast cells (Figure 5).

The 72 h cultures on blood agar and Sabouraud agar were subcultured on blood and Sabouraud agar, respectively. After 24 h, the growth on both solid media was weak and barely perceptible, and after 48 h, the growth was visible and more prolific on the Sabouraud dextrose agar.

Three days after the first thoracentesis, the patient was brought in again with tachypnea (56 min^−1^) and tachycardia (187 min^−1^) but with a normal rectal temperature (38 °C) and CRT (2 s). The radiography confirmed the presence of fluid by the partial border effacement of the cardiac silhouette (Figure S2), so a second thoracentesis was performed only on the left side in the 8th intercostal space using the same anaesthesia protocol. The evacuated pleural fluid was 160 mL, white, opaque, odourless, and viscous. On the post-thoracentesis radiograph taken for evaluation of the condition of the cat, the heart was better visualised, but some pleural fluid still remained in the cranioventral part of the chest. The blood analysis showed leukocytosis (31.9 G/L; reference range 5.5–19.5 G/L) with neutrophilia (23.7 G/L; reference range 2.5–12.9 G/L), but less severe compared to the first blood tests. The owners were warned about the possibility of repeated pleural fluid accumulation.

On the basis of the preliminary results from the microbiological examination suggesting the involvement of yeasts, treatment with 40 mg/kg terbinafine (Terbinafin 250 mg, Genericon Pharma GmbH, Graz, Austria), half a tablet daily for 15 days, was prescribed. The antifungal therapy was complemented with furosemide (Furosemid 5%, Alfasan, Woerden, Netherlands) at 2.5 mg/kg subcutaneously, clindamycin (Clindavet 75 mg, Provet, Attiki, Greece) at 5.5 mg/kg every 12 h, and Ringer lactate (Ringer Braun, B.Braun Melsungen AG, Germany) at 10 mL/kg per hour.

After the presumptive morphological testing, the isolate was submitted for matrix-assisted laser desorption ionisation–time of flight mass spectrometry (MALDI-TOF MS) identification at the National Center of Infectious and Parasitic Disease (Sofia, Bulgaria). The peaks generated by the Bruker MALDI-TOF MS were matched to reference libraries using the BioTyper software v. 4.1.100 (Bruker Daltonics, Bremen, Germany). The isolate was identified as *Clavispora lusitaniae*—NCBI:txid36911; best match score 2.27; second best match 2.13, corresponding to highly accurate yeast identification [12].

Three days later, a third left-sided thoracentesis was deemed necessary based on the obscured heart silhouette for the evacuation of 190 mL of milky white fluid. On the follow-up right lateral radiograph, the cardiac silhouette and the precordial space were already visible (Figure S3). After the procedure, the clinical status of the cat was improved. The microbiological examination of the pleural fluid sample from the third thoracentesis was repeated; the direct inoculation isolated only a single colony of the pathogen on the blood agar plate after 48 h incubation.

After the 15-day treatment course, the owners informed us that the patient was active and had regained its appetite. A follow-up examination of the patient was scheduled 15 days after the end of the antifungal treatment. The general condition of the patient was very good, with normal respiratory and heart rates. The blood analysis demonstrated normal total leukocyte (8.21 G/L; reference range 2.5–12.9 G/L) and neutrophil counts (3.06 G/L; reference range 2.5–12.9 G/L), as well as slightly increased eosinophil counts (0.95 G/L; reference range 0–0.8 G/L). Serum creatine kinase was mildly elevated (255 U/L; reference range 70–160 U/L). The blood globulin concentration (43 g/L) was within the reference range (28–45 g/L).

**Figure 6:**
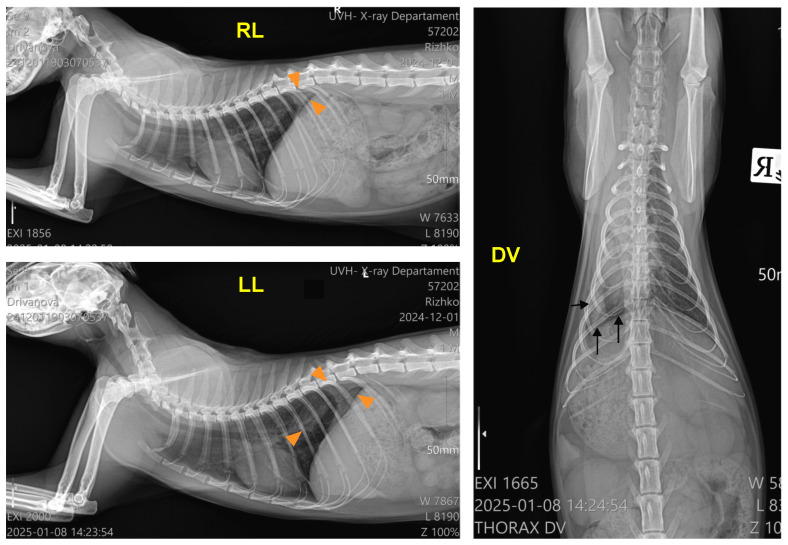
Right lateral (RL), left lateral (LL), and dorsoventral (DV) radiographs of the patient during the control examination 15 days after the end of antifungal therapy: areas of moderate atelectasis (arrowheads on RL and LL views); uniform radiopacity in the area of the costophrenic angle (black arrows; DV view).

On control lateral radiographs (Figure 6), the cardiac silhouette was already clearly seen. Confined dense focal uniform shadows (atelectasis) were observed in the caudodorsal part of the lungs. The dorsoventral view demonstrated a uniform radiopacity in the area of the costophrenic angle, with retraction of the lung lobe (on the left side). The trachea was not displaced, the heart was visible, and moderate atelectasis was present.

## 3. Discussion

Pleural effusion is a life-threatening condition with progressive worsening dyspnoea and substantial risk of a fatal outcome [13]. The differential diagnoses of thoracic pleural effusion in cats includes congestive heart failure, pyothorax, chylothorax, neoplasia, feline infectious peritonitis, and trauma [14]. In this case, the definitive diagnosis was pyothorax, as the three criteria for fungal empyema thoracis outlined by Ko et al. [15] were met: (1) isolation of a fungal species from the pleural effusion; (2) significant signs of infection, such as fever and leukocytosis; and (3) isolation of the same fungal agent from pleural effusion on more than one occasion, or from pleural effusion and other specimens.

The septic pleural fluid is usually opaque with flocculent matter, creamy, green-tinged, or sanguineous. In our patient, a chylous effusion was initially suspected, because the fluid was odourless, but this was excluded due to the low triglyceride content (0.4 mmol/L). The lack of odour does not rule out an infectious cause, as 20% of cases of infectious pleuritis, particularly in kittens, are caused by unusual bacterial, fungal, or protozoal pathogens [14].

Pyothorax is a progressive illness. The disease may be present 1–2 weeks to a month before the specific clinical signs appear and the effusion becomes extensive [16]. In the presented patient, the owners had noticed changes in the usual behaviour of the pet (lethargy, decreased appetite) for 2 to 3 weeks, and clinical signs (accelerated breathing and cough) appeared 7 days before the referral.

The underlying cause of pyothorax in cats often remains unknown [1,17]. Penetrating injuries, such as bite wounds, as a cause for pyothorax are supported by the findings that outdoor cats in multi-cat households are commonly affected, but a parapneumonic spread has also been suggested as a frequent cause [4]. In our patient, a visible entrance door of infection was not identified, as it only had cicatrices from past injuries, which may be a possible etiological cause in line with the cited literature data.

The observed changes in blood parameters agree with those that are commonly reported in cats with pyothorax. Leukocytosis, neutrophilia, and monocytosis are the most important reported CBC changes [2]. Mild-to-severe hyperglubulinaemia is also often seen in cats with IFIs [6]. The significantly increased serum creatine kinase activity at referral may be attributed to prolonged anorexia and weight loss. In anorexic cats, Fascetti et al. [18] reported elevated creatine kinase activity from moderate to more than 150,000 IU/L (reference 10–100 IU/L).

The morphology of fungal colonies and microscopy of fungal colony smears are usually sufficient for identification at the genus level. PCR or MALDI-TOF mass spectrometry of cultured fungi are the most reliable methods for definitive identification of the IFI pathogen.

Scarce data are available for the role of *C. lusitaniae* as a pathogen in animals. As already stated, invasive infection with *C. lusitaniae* in a cat has been reported only in a peritonitis case [10]. *C. lusitaniae* was histologically proven in a pneumonic lung sample from a sea turtle [19]. In India, the analysis of swab samples from dogs with confirmed otitis externa demonstrated that the ear skin of one dog was colonised with multiple yeast species, including *C. lusitaniae*. The same yeast species was isolated from the skin microflora of two other healthy dogs [20].

The most common source of yeast infections is the environment. *C. lusitaniae* has been isolated from a variety of substrates: water, soil, fruits, and vegetables [21]. Several decades ago, this yeast species was recovered from rock pigeon faeces near Turin, Italy [22], along with *C. guilliermondii*, *C. albicans*, *C. parapsilosis*, and *C. rugosa*, many of which are considered pathogenic. Another study confirmed pigeons as main reservoir of different yeast species of public health importance and their faeces as an ideal environment of yeast replication. *C. albicans* was the most frequent isolate from faecal samples collected in public squares (29.4%), followed by *C. krusei* and *C. inconspicua* (17.6%). *C. lusitaniae*, *C. glabrata,* and *C. pelliculosa* have been detected only in one public square (5.9%) [23]. Taking into consideration the very large population of pigeons in modern cities, it may be suggested that their faeces are a very probable source of contamination with yeasts for dogs and cats with outdoor access, as was the case with our patient.

So far, no study has investigated the interaction between *C. lusitaniae* and its host. It could be hypothesised that one possible virulence factor is thermotolerance, as it replicates in hosts with body temperatures that are higher that the optimal for fungal growth [9]. Data from the literature have proven the ability of *C. lusitaniae* to form biofilm due to its hydrophobic capacity [24,25]. Biofilm formation by *Candida* spp. is important for adhesion to surfaces, protection of the fungal cells against chemical agents, and particularly for host immunity evasion. Garcia-Perez et al. [26] proposed three basic models to explain the latter phenomenon: (1) immunological silence that “hides” the biofilm from the host immune system, which fails to activate its anti-*Candida* mechanisms; (2) immune deviation, in which biofilm-produced factors make the immune response ineffective; and (3) immune resistance, comprising the structural or molecular biofilm features that make it resistant to immune clearance, even in the presence of an effective biofilm-induced response.

After the initial referral of the patient to the clinic, the broad-spectrum lincosamide clindamycin was prescribed empirically to control the most commonly encountered agents of feline pyothorax—anaerobes and Gram-positive aerobes (including most *Staphylococcus* spp. and *Streptococcus* spp.) [27]. As only a few antifungal medications are licenced for veterinary species, the choice of terbinafine was based mostly on its activity spectrum and the convenient tablet size. Terbinafine has been administered in cats at 30–40 mg/kg orally once a day [28]. It is active against many fungi, including yeasts (for example, *Candida*, *Cryptococcus*, *Malassezia*), as well as dermatophytes. In addition to the fungicidal activity, terbinafine possesses anti-inflammatory action due to its scavenging properties [29]. The duration of antifungal treatment is greater than that of therapy with antibiotics, because of the slow growth rate of fungi and the fungistatic mechanism of action [30]. After the treatment course, the condition of the patient improved, and it remained free of clinical signs for 15 days after stopping the terbinafine.

## 4. Conclusions

The described case emphasises the primary importance of timely identification of pathogenic agent(s) of feline pyothorax and appropriately prescribed treatment for the prevention of severe complications and fatal outcomes. Although most known invasive fungal infections in cats are not zoonoses, the close bond between humans and pets confers a real risk from direct transmission of emerging yeast species that may cause dangerous fungal infections, especially in immunocompromised people.

## Data Availability

Data are contained within the article and Supplementary Materials.

## Supplementary Materials

The following supporting information can be downloaded at https://www.mdpi.com/article/10.3390/jof11030170/s1: Figure S1: Right lateral radiograph of the patient after the first thoracentesis: improved visualisation of the heart (black arrowheads) and a small amount of pleural fluid (orange arrowheads); Figure S2: Right lateral radiograph of the patient before (left) and after (right) the second thoracentesis; Figure S3: Right lateral radiograph of the patient before (left) and after (right) the third thoracentesis. Following the procedure, the heart and precordial space (arrowheads) were already visible.
