# Supplementary material for: Fungal Empyema Thoracis Associated with Clavispora lusitaniae—First Report in a Domestic Cat
Source: J Fungi (Basel). 2025 Feb 20;11(3):170. doi: 10.3390/jof11030170 (PMC11942772; doi:10.3390/jof11030170)
Supplement: Supplementary file 1 [file jof-11-00170-s001.zip › jof-3452090-supplementary.pdf]

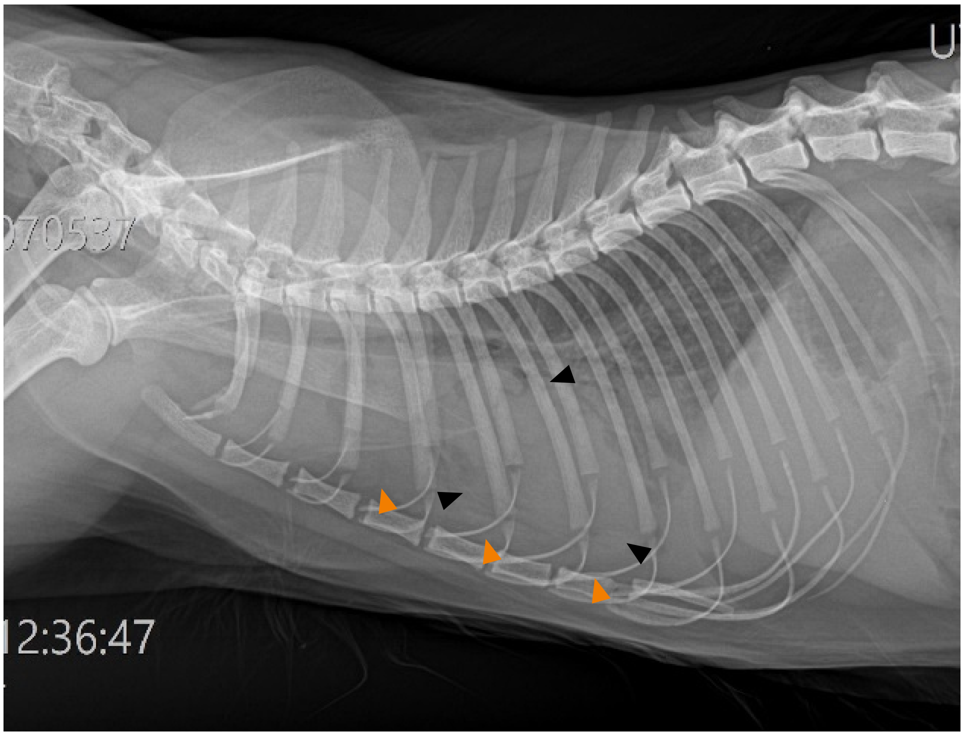

Figure S1. Right lateral radiograph of the patient after the first thoracentesis: improved visualisation of the heart (black arrowheads) and a small amount of pleural fluid (orange arrowheads).

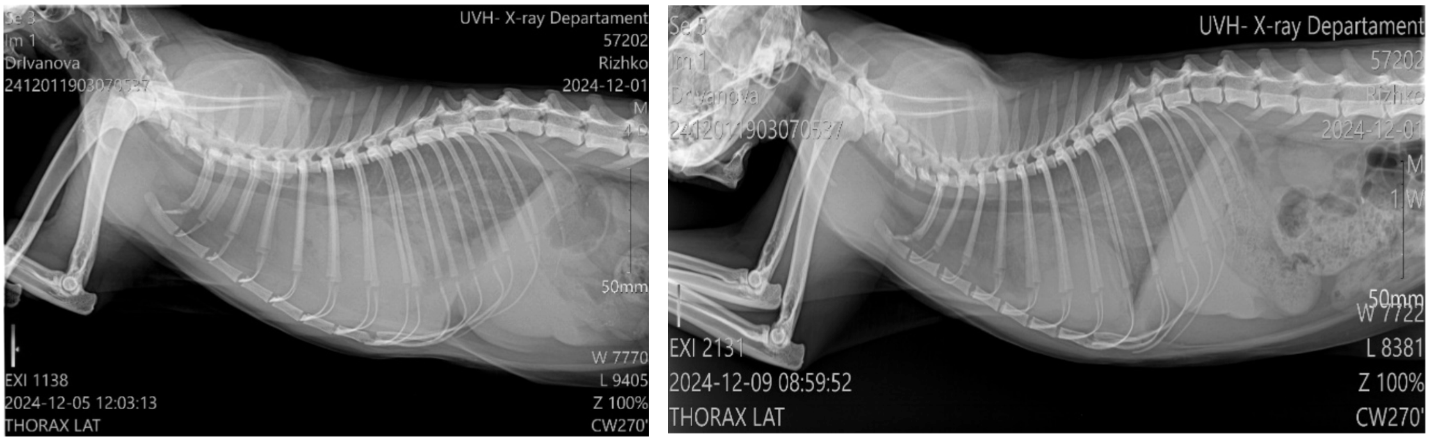

Figure S2. Right lateral radiograph of the patient before (left) and after (right) the second thoracentesis

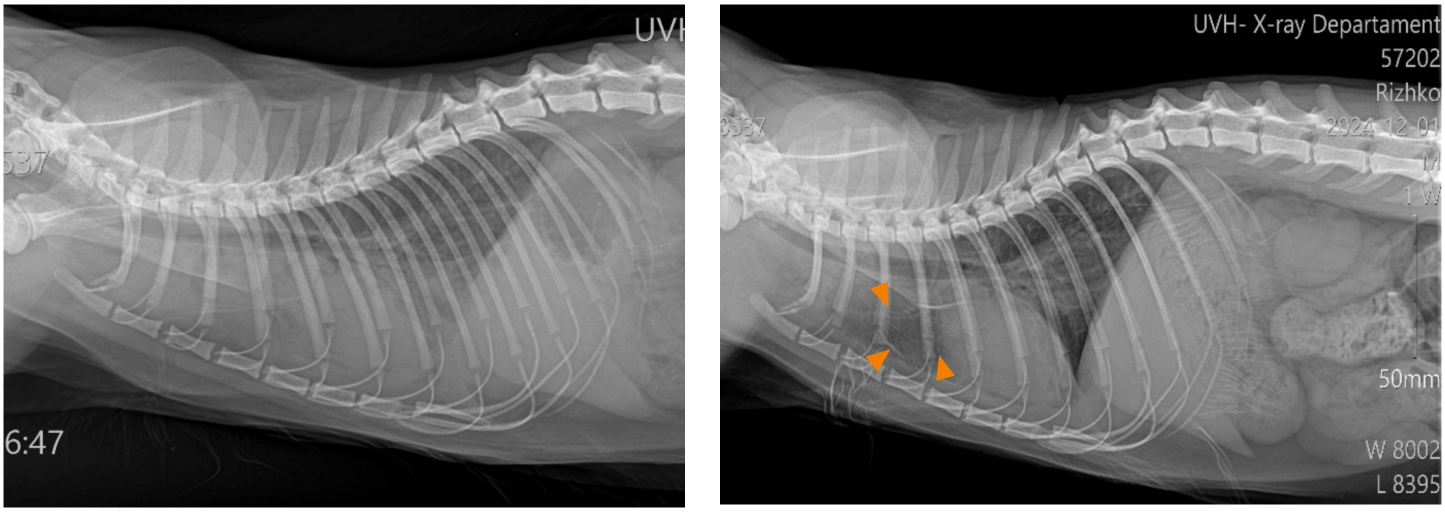

Figure S3. Right lateral radiograph of the patient before (left) and after (right) the third thoracentesis. Following the procedure, the heart and precordial space (arrowheads) were already visible.
